# Prevalence of diarrhea and associated factors among under-five children in Bahir Dar city, Northwest Ethiopia, 2016: a cross-sectional study

**DOI:** 10.1186/s12879-019-4030-3

**Published:** 2019-05-14

**Authors:** Amare Belachew Dagnew, Tilahun Tewabe, Yihun Miskir, Tariku Eshetu, Wosin Kefelegn, Kidanu Zerihun, Mekonnen Urgessa, Tiruha Teka

**Affiliations:** Bahir Dar University, College of Medicine and Health Sciences, Bahir Dar, Ethiopia

**Keywords:** Diarrhea, Under -five children, Bahir Dar, Ethiopia

## Abstract

**Background:**

In Ethiopia, morbidity and mortality due to diarrhea is significantly high. Most importantly, burden of diarrhea is disproportionately high among under-five children. Therefore, the objective of this study was to assess the prevalence and factors associated with diarrhea among children younger than 5 years old in Bahir Dar city, Northwest, Ethiopia, 2016.

**Methods:**

This community-based cross-sectional study was conducted among under-five years-old children from March 24 to April 12, 2016. Systematic sampling technique was used to select 498 households. Data were collected by using an interviewer administered questionnaire. Both bivariate and multivariate logistic regression analyses were employed to identify predictor variables. Factors with a *p*-value of < 0.05 were considered as independently associated with diarrhea.

**Results:**

The 2 weeks prevalence of diarrhea among under five children was 14.5%. Lack of hand washing facilities in the household (AOR = 3.910 (1.770, 8.634)), lack of separate feeding materials (AOR = 5.769 (1.591, 9.220)), poor hand washing practice (AOR = 6.104 (2.100, 17.738)) and not breastfeeding (AOR = 2.3 (1.023, 5.46)) were predictors of the concurrence of diarrhea.

**Conclusions:**

The prevalence of diarrhea in the study area was slightly higher than the 2016, Ethiopian Demography and Health Survey finding which was 12%. Thus, improving handwashing facilities and practices, serving the food to the child with a separate materials and encourage optimal breastfeeding were recommended.

**Electronic supplementary material:**

The online version of this article (10.1186/s12879-019-4030-3) contains supplementary material, which is available to authorized users.

## Background

Diarrhea is thepassage of three or more watery or loose stools per day, and when the mother considered as increased stool frequency or liquidity [1]. The acute diarrhea causes high loss of water and salts from a body which results in either severe dehydration and death within a short period of time or predisposes children to malnutrition and makes them more susceptible to other infections [2].

Globally, around 1.3 million people were died due to diarrhea and of the total deaths about 0.54 million were children’s of under-five years of age. Majority of the mortality were occurred in developing countries [3]. Diarrhea is the second most cause of mortality and morbidity of under-five childhood illnesses in sub-Saharans Africa countries. In these countries, only 40% of people living in urban setting were accessing improved sanitations [3] and 72% of the people in Ethiopia were living without improved sanitation facilities [4].

Diarrhea is a highly prevalent disease in Ethiopia. In fact, it is the second leading cause of deaths [2, 3]. A previous 2 week survey showed that prevalence of diarrhea among children under 5 years old was 13% [3].

Different studies in Ethiopia showed that, socioeconomic status, monthly income, number of under-five children, methods of complementary feeding, types of water storage equipment, mother’s poor hand washing practices, lack of hand washing facilities, duration of breastfeeding and improper waste disposal practices were significant factors for diarrhea occurrences [5–9].

Identifying the contributing factors of diarrhea is very important for the effective implementation of child health programs and prioritizations. There is a considerable variation in prevalence and determinant factors for diarrhea occurrence in Ethiopia. Despite the interventions and innovations undertaken regarding childhood diarrhea, the burden of the disease is high in the current study area. Therefore, this study was conducted to assess the magnitude and associated factors of diarrhea among under 5 years old children in Bahir Dar city, North West, Ethiopia.

## Methods

### Study participants and period

A community based cross sectional study was conducted among under five children from 24 March to 12 April, 2016, in Bahir Dar city, Amhara Regional State, Ethiopia. Bahir Dar city has nine sub cities and 18 kebeles. The current population size of the city is 318,429. There are two public hospitals, two private hospitals and 9 health centers in the city.

### Sample size determination and sampling techniques

The sample size was calculated using a single population proportion formula with consideration of the following assumptions: Prevalence (P) = 18% which is the prevalence of diarrhea in Mecha district, North West Ethiopia [10], confidence level (CL) 95%, margin of error (d) =5%, design effect = two (Bahir Dar city- sub city –kebeles- households) and 10% non- response rate. Thus, the final calculated sample size was 499. Household numbers having under-five children were taken from health extension workers registration books. The sampled households were proportionally allotted to each selected kebeles. Systematic sampling technique was used to select households from each kebele. The first household was selected using the lottery method among fife households, while the rest households, were selected every fifth interval.. The youngest child was selected for a household having two or more under 5 years of children.

### Data collection tools and procedures

Data were collected from mothers/primary care takers using an interviewer-administered questionnaire and observation checklists. The questionnaire was adapted from previous research done on a similar topic [5, 6, 10] and customized accordingly. A two-week recall method was used to assess the prevalence of diarrhea. Data collectors and supervisors were recruited for data collection. The supervisors and data collectors were trained about interview methods, consent and ethical aspects during data collection.

### Statistical analysis

The questionnaire was checked visually for completeness and coding was given. The data was entered into Epi-info version 3.5.4 and exported to SPSS version 21.0 software for analysis. Frequency tables were used to summarize the socio-demographic characteristics’ of the study participants and magnitude of diarrhea. Then, bivariate logistic regression was performed for each independent variable with the outcome variable and those variables with a *p*-value of < 0.05 were adjusted into the final model (multivariate analysis). Finally, those variables having a *P* value less than 0.05 were declared as statistical significant.

### Operational definition

Diarrhea: The occurrence of loose or watery diarrhea at least three times per day in the previous 2 weeks, as reported by the mother/caretaker of the child [5].

Mixed feeding: An infant who took both breast milk and other food or liguid before his/her six months of age.

## Results

### Socio-demographic-characteristics

Of 499 households, 498 were included in this study with a response rate of 99.8%. More than half of mothers’ age (54.8%) was between in the age group of 25 and 34 years with the mean age of 28.94 years (± 7.61 SD). Two hundred five (41.2%) mothers attended primary level of education and 55.2% were housewives by ocuupations. The mean family size of the households was 4.43 (±1.37 SD) persons, meanwhile most (86.3%) of the house-hold had only one under-five children in the family [Table 1].

**Table 1:** Socio-demographic characteristics of households in BahirDar City, Amhara region, Ethiopia, 2016

Variables Name	Category	Frequency(*n* = 498)	Percent
Household family size	Four and less	304	61.0
	More than four	194	39.0
Number of under five children in the house	One child	430	86.3
	Two and above	68	13.7
Relation of respondents to the child	Mother	385	77.3
	Caretaker	113	22.7
Age of mothers	18–24	130	26.1
	25–34	273	54.8
	35 and above	95	19.1
Marital status of mothers	Married	386	77.5
	Others	112	22.5
Religion	Orthodox	415	83.3
	Others	83	16.7
Occupation of mothers	Employed	73	14.7
	Housewives	275	55.2
	Others	150	30.1
Education status of mothers	No formal education	110	22.1
	Primary	205	41.2
	Secondary and above	183	36.7
Education of fathers	No formal education	41	8.2
	Primary	95	19.1
	Secondary and above	362	72.7
Occupation of fathers	Government employee	192	38.6
	Merchant	227	45.6
	Others	79	15.9

### Environmental characteristics of the households

Two hundred eighty-six (57.4%) and 477 (95.8%) of the households had the dwelling with the mud floor and corrugated iron roof, respectively. A majority of the households had a latrine and hand washing facility which accounts 96.4 and 60.2%, respectively. About 64.2% of households had private latrine and from them 30.6% were not improved in type. Most households (95.6%) disposed their waste materials properly. All households were pipe water users and most (78.3%) of the households consumed more than 20 l water per day [Table 2].

**Table 2:** Environmental characteristics of the households in Bahir Dar city, Amhara region, Ethiopia, 2016

Name of variables	Category	Frequency (n = 498)	Percentage
Household floor type	Mud	286	57.4
	Cement	212	42.6
Household roof type	Corrugated iron	477	95.8
	Others	21	4.2
Hand wash facilities	Yes	300	60.2
	No	198	39.8
Latrine availability	Yes	480	96.4
	No	18	3.6
Type of latrine (*n* = 480)	Improved	333	69.4
	Not improved	147	30.6
Owner ship of latrine (n = 480)	Private	308	64.2
	Shared	172	35.8
Faces seen in the pit hole (n = 480)	Yes	52	10.8
	No	428	85.9
Faces seen around the compound (n = 498)	Yes	34	6.8
	No	464	93.2
If no latrine where they use(*n* = 18)	Open field	16	88.9
	Others	2	11.1
Refuse disposal method (n = 498)	Proper	476	95.6
	Improper	22	4.4
Water storage container	Jerry can	360	72.3
	Others	138	27.7
Mean daily water consumption 41.94 ± 17.43SD			

### Behavioral characteristics of the respondent

Most (81.1%) of the respondents practiced mixed feeding. More than one-third (37.6%) of respondents had fed cow’s milk to their children. About 88.6% of caregivers reported that they wash their hand using soap with water. However, one-fourth of caregivers didn’t wash their hands after cleaning the child.On the other hand more than three fourth (74.8%) of respondents used a cup and spoon to feed their children [Table 3].

**Table 3:** Behavioral characteristics of the respondents in Bahir Dar city, Amhara Region, Ethiopia, 2016

Variable type		Category	Frequency (*n* = 498	Percentage
The child take other food than breast feed		Yes	404	81.1
		No	94	18.9
Type of food the child take mostly (*n* = 404)		Cow’s milk	152	37.6
		Gruel	118	29.2
		Adult food	44	10.9
		Others	90	22.3
Child feed method (n = 404)		Hand	53	13.1
		Cup &spoon	302	74.8
		Bottle	49	12.1
Hand washing	Before food preparation	Yes	377	75.7
		No	121	24.3
	Before eating	Yes	483	97
		No	15	3
	After visiting latrine	Yes	447	89.8
		No	51	10.2
	After cleaning child’s bottom	Yes	377	75.7
		No	121	24.3
Hand washing method		Water & soap	441	88.6
		Water only	57	11.4

### Demographic and health characteristics of the indexed children

More than half (53.8%) of the children were males and the mean age of the children was 21.07(±14.4 SD) months. Almost all of the children received measles and Rotavirus vaccine which is (97.9%) and (97.1%), respectively. Seventy two (14.5%) children had diarrhea during the 2 weeks at the time of data collection [Table 4].

**Table 4:** Demographic and health characteristics of the indexed children in Bahir Dar City, Amara region, Ethiopia, 2016

Name of variables	Category	Frequency(*n* = 498)	Percent
Age of Child (in month)	Eleven month and less	145	29.1
	12–23	152	30.5
	23–34	98	19.7
	Greater than 35	103	20.7
Sex of Child	Male	268	53.8
	Female	230	46.2
Duration of Breast Feeding	Less than 24 months	403	76.5
	Greater than or equal to24 months	95	23.5
Current Breast Feeding Status	Exclusive Breast Feeding	83	16.7
	Partial Breast Feeding	289	58.0
	No Breast Feeding	126	25.3
Age at complementary Feeding	Less than Six Month	94	18.8
	At Six Month	355	71.2
	Greater than Six month	49	10
Measles Vaccine(n498)	Yes	484	97.1
	No	14	2.9
Rota Virus Vaccine(n = 498)	Yes	484	97.1
	No	14	2.9
Prevalence of Diarrhea	Yes	72	14.5
	No	426	85.5
Mean age of children in months 21.07 ± 14.4SD			

### Factors associated with childhood diarrhea

Bivariate analysis was done and variables having a (*P* value of leass than < 0.05) were enrolled to the multivariable analysis for association testing. Those variables having a *p* value of less than 0.05 were declared as significantly associated with diarrhea.

Children living in households with no hand washing facilities were around four times more likely to develop diarrhea than their counterparts ([AOR = 3.91(1.770, 8.634)]. Additionally, Children whose mother used only water to wash their hand were six times more likely to develop diarrhea than children whose mother used water with soap to wash their hands [AOR: 6.104 (2.100, 17.738)].

Regarding feeding practices of the child, non exclusive breastfed children were 2.3 times more likely to develop diarrhea than children who fed breast milk only during the first 6 months of life [AOR: 2.3 (1.023, 5.46) [Table 5]. Again, children who did not use separate feeding materials were six times at high risk to develop diarrhea than children who used their own material for feeding [AOR = 5.769 (1.591, 9.220)] [Table 5].

**Table 5:** Multivariate analysis on determinants of under-five diarrhea in Bahir Dar City, Amhara region, Ethiopia, 2016

Name of Variables	Category	Yes	No	COR (95% C.I)	AOR (95% C.I)
Hand wash facilities	No	46(23.2)	152(76.8)	3.189(1.896,5.365)	3.910(1.770,8.634)
	Yes	26(8.7)	274(91.3)	1	
Separate material for the child feeding	No	20(45.5)	24(54.5)	4.936(2.546,9.571)	5.769(1.591,20.920)
	Yes	52(14.4)	308(85.6)	1	
Ownership of Latrine	Shared	32(18.6)	140(81.4)	2.28(1.324,3.947)	
	Private	28(9.1)	280(90.9)	1	
Type of Latrine	Not improved	35(23.8)	112(76.2)	3.850(1.206,6.720)	
	Improved	25(7.5)	308(92.5)	1	
Hand washing practice	Water Only	21(36.8)	36(63.2)	4.461(2.418,8.228)	6.104 (2.100,17.738)
	Water and soap	51(11.6)	390(88.4)	1	
Breastfeeding	No BF	23(18.3)	103(81.7)	5.955(1.727,20.537)	2.3(1.023, 5.46)
	Partially BF	46(15.9)	243(84.1)	5.048(1.528,16.675)	
	EBF	3(3.6)	80(96.4)	1	
Faces around the compound	No	59(12.7)	405(87.3)	4.249(2.020,8.939)	
	Yes	13(38.2)	21(61.8)	1	

## Discussion

The two weeks prevalence of childhood diarrhea among under-five years-old in this study was 14.5%. This finding is higher than studies done in East Gojjam zone, 6.5% [11] and EDHS 2016 report, 12% [12]. However, the current result is lower than west Gojjam zone, 18% [10], Egypt, 19.5% [13], Ghana, 19.2% [14], Eastern Ethiopia, 22.5% [8], Iraq, 21.3% [15], India 25.2% [16] Burundi, 32.6% [17], Arba-Minch rural community, 31% [18], and Benshangul gumz, 22.1% [19]. This may be due to; the current study setting consider only urban dwellers; differences in socio-demographic characteristics, environmental factors (climate and geographical differences) and behavioral factors (availability of water, presence and usage of latrine, availability of hand washing facilities, ways of waste disposal and food storage mechanisms in the study area compared to other studies.

Children living in households without hand washing facilities were 3.9 times more likely to develop childhood diarrhea compared to households with hand washing facilities. This finding is in line with studies done in Ghana [14], Benshangul Gumz [19] and East Gojjam [11]. This may be due to the fact that using improved latrine and presence of a hand washing facility decreases the contamination of foods and water by pathogenic organisms. Similarly, in the current study, children whose mother used only water to wash their hand were six times more likely to develop childhood diarrhea than children whose mother used water with soap to wash their hand. This finding is in line with studies done in south-west Ethiopia [20], Jijjiga [21] and Bangladesh [22]. This might be due to soap has antimicrobial agent, and it is effective to prevent diarrhea and occurrence of other hygiene related diseases. Proper hand washing can eliminate microorganisms.

Mothers feeding practices is a vital indicator for a child health. In this study, diarrhea was significantly associated with breastfeeding practices, non exclusive breastfed children were 2.3 times more likely to develop diarrhea than exclusive breastfed children during the first 6 months of their life. This finding is consistent with systematic review studies done in globe [23] and United States [24]. Which could be due to breast milk has its own polysaccharide immunoglobine A that prevents gastroenteritis by coating thegastrointestinal tract. In addition to this, breast milk is natural, safe, appropriate, cheapest and free of contamination from microbes [25]. Therefore, encouraging exclusive breastfeeding for the first 6 months of life is very important to reduce child morbidities and mortalitiesy. On the other hand, the current finding also showed that children who did not use separate feeding material were 5.7 times more likely to develop diarrhea than their counterparts which is in line with studies done in Debre Birhan [26] and Nigeria [27]. The possible reason might be, the materials that were used in common with other family member might be contaminated. Beyound this, caregivers may neglect cleaning of feeding materials that they used for serving food to their child.

## Conclusions

Considering the current study area is urban setting, the prevalence of childhood diarrhea was high. Lack of hand washing facilities, lack of separatly feeding materials for children, not breastfeed exclusively and hand washing practice were predictors of occurrence of diarrhea. Thus, improving handwashing facilities and practices, serving the food to the child with a separate materials and encourage optimal breastfeeding were recommended.

## Additional file

Additional file 1: English questionaire of diarrhea. (DOCX 43 kb)
