# Supplementary material for: Prevalence of diarrhea and associated factors among under-five children in Bahir Dar city, Northwest Ethiopia, 2016: a cross-sectional study
Source: BMC Infect Dis. 2019 May 14;19:417. doi: 10.1186/s12879-019-4030-3 (PMC6518740; doi:10.1186/s12879-019-4030-3)
Supplement: Supplementary file 1 — English questionaire of diarrhea. (DOCX 43 kb) [file 12879_2019_4030_MOESM1_ESM.docx]

**ANNEXS-I**

**Annex I: English questionnaire**

Household Identification Code______________

Name of Kebele______________

**Annex A: Verbal Consent Letter**

Dear interviewee, I extend my greeting to you. I / we are here to collect health related data for the purpose of research from Bahir Dar university, College of Medicine& Health Science, Department of Nursing. The aim of this study is to assess the prevalence of diarrhea and associated factors among children under five years in Bahir Dar city. We are requesting your permission to participate in an interview on issues related to the prevalence of diarrhea and its associated factors.

This information will help the policy makers and other responsible bodies as background to +improve the health status of the children related to acute diarrhea .

We assure you that whatever information you provide will only be used for the purpose of this research and will not be made available to anyone outside of the research team.

Your willingness and support to people respond the interview is very much appreciated. We also assure that the interview process will not bring any harm to you and your family. It is also your right to withdraw any time from the process when your feeling is uncomfortable with it.

**Please make (X) mark to indicate the respondents’ decision regarding participation in the study.** The purpose of the study and confidentiality procedures has been explained to me and I on my own consent: a) Agree _____ b) Disagree_____

Interviewer name______________________ Signature______________________

Date of interview_______________Time started__________ Time completed________

Result of interview: 1. Completed 2. Respondent not available

3. Refused 4. Incomplete

Checked by supervisor: Name____________________ Signature_______ Date_______

**Questionnaire for the prevalence of diarrhea and associated factors**

### Part one: Socio-Demographic characteristics

Q1. Name of kebele----------------------

Q2. Number of persons in the household---------------

Q3. Number of under-five children in the household--------------

Q4. Relation of the respondent to the child

1. Mother

2. Caretaker

Q5. Age of the mother/caretaker---------------years

Q6. Marital status of the mother/caretaker

1. Married

2. Divorced

3. Single

4. Widowed

Q7. Religion of parents/caretaker

1. Christian

2. Muslim

3. Protestant

4. Other (specify)___________

Q8. Educational status of the mother/caretaker

1. Unable to read and write

2. Able to read and write

3. Primary

4. Secondary and above

Q9. Occupational status of the mother/caretaker

1. Government employee

2. Housewife

3. Merchant

4. Farmer

5. Others (specify)___________

Q10. Educational status of the father

1. Unable to read and write
2. Able to read and write
3. Primary
4. Secondary and above

Q11. Occupation of the father

1. Government employee

2. Merchant

3. Farmer

4. Daily labour

5. Others (specify )_______

### Part two: Environmental health conditions

Q1. Type of floor material of the living house(observation)

1. Mud

2. Wood

3. Cement

4. Other (specify)____________

Q2. Type of roof material of the living house(observation)

1. Wood

2. Thatched

3. Corrugated iron sheet

4. Others (specify)__________

Q3.Do you have hand washing facility?

1. Yes

2. No

Q4. Do you have latrine?

1. Yes

2. No

Q5. Type of latrine facility (observation)

1. Flush/pour flush to piped sewer system

2. Flush/pour flush to septic tank

3. Flush/pour flush to pit latrine

4. Ventilated improved pit (VIP) latrine

5. Pit latrine with slab

6. Pit latrine without slab

7. Others (specify)_________

Q6. Ownership of the latrine

1. Private

2. Shared with neighbors

Q7. Is feces seen around the pit hole (floor)?(observation)

1. Yes

2. No

Q8. Is feces seen around the house (compound)?(observation)

1. Yes

2. No

Q9. If the household has no latrine, where do you dispose human waste?

1. Open field

2. Other (specify)___________

Q10. How do you dispose refuse?

1. Pit

2. Open field

3. Burning

4. Garbage can

5. Other

Q11. Type of collection container

1. Pot

2. Plastic bucket

3. Iron bucket

4. Jerry can

5. Other_____________

Q12. Source of water supply_________

Q13. Daily requirements of waters in litters.___________

**Part three: Behavioral conditions**

Q1**.** Does the child take other food than breast milk?

1. Yes

2. No

Q2. Do you separately prepare food using separate material for the child?

1. Yes

2. No

Q3. What food/fluid is the child mostly taking (if the child is not on exclusive breastfeeding)?more than one is possible

1. Cow’s milk

2. Powder milk3. Gruel

4. Adult food

5. Other (specify)___

Q4. What do you use to feed the child?

1. Hand 3. Bottle

2. Cup and spoon 4. Others__________

Q5**.** In which condition did you wash your hand?

1. Before food preparation and eating

2. After eating

3.After visiting latrin

4. After cleaning of child bottom

5. other (specify)________________

Q6. What did you use to wash your hands?

1. Soap& water

2. Ash & water

3. Only water

4. Others (specify)_______

### Part four: Information of the index child

Q1. Age-------------Months

Q2. Sex -------------

Q3. For how long did you breastfeed your child? -------------- Months

Q4. What is his/her current breast feeding status?

1. Exclusive breast feeding

2. Partial breast feeding

3. Not breast feeding

Q5. At what age did the child start supplementary feeding/weaning food? ------------Months

Q6. Did the child receive measles vaccination (for those greater than nine months age)?

1. Yes

2. No

Q7. Did the child receive Rota virus vaccination? (Rvv1, Rvv2)

1. Yes

2. No

Q8. Does your child experience frequent and copious discharge of abnormally liquid faeces or diarrhea within the last two weeks?

1. Yes

2. No .
